# A Review of the Application of Virtual Reality Technology in the Diagnosis and Treatment of Cognitive Impairment

**DOI:** 10.3389/fnagi.2019.00280

**Published:** 2019-10-18

**Authors:** Yao Liu, Wenjun Tan, Chao Chen, Chunyan Liu, Jinzhu Yang, Yanchun Zhang

**Affiliations:** ^1^Key Laboratory of Intelligent Computing in Medical Image, Ministry of Education, Northeastern University, Shenyang, China; ^2^Cyberspace Institute of Advanced Technology, Guangzhou University, Guangzhou, China; ^3^Key Laboratory of Complex System Control Theory and Application, Tianjin University of Technology, Tianjin, China; ^4^Department of Neurology, Xuanwu Hospital, Capital Medical University, Beijing, China; ^5^Centre for Applied Informatics, College of Engineering and Science, Victoria University, Melbourne, VIC, Australia

**Keywords:** virtual reality, cognitive impairment, cognitive training, cognitive rehabilitation, Alzheimer’s disease

## Abstract

At present, with the development of an aging society and an increase in the number of elderly people, in order to ensure the ability and enthusiasm of the elderly to live independently, it is necessary to ensure that they can understand the world in a normal way. More and more elderly people have cognitive impairment, and virtual reality (VR) technology is more effective in cognitive diagnosis and treatment than traditional methods. This review article describes some studies on cognitive diagnosis and training for the elderly, and puts forward some suggestions for current studies, in the hopes that VR technology can be better applied to cognitive diagnosis and training.

## Introduction

### Cognitive Impairment

Cognitive processes are defined by a neural process that allows us to interact with complex environments in a goal-oriented manner. It is a multifaceted process that includes attention, memory, language, emotion, etc. When multitasking is done at the same time, the normal brain can correctly process the information obtained from the complex environment and avoid interference with useless information. When the brain is in a normal state, this process can proceed smoothly, but if the brain produces lesions, this cognitive process will fail, leading to memory impairment, learning disabilities, attention deficits, and aphasia (Anguera et al., 2013).

Moreover, with the aging of today’s society, age is the most common cause of cognitive impairment in an increasing number of people entering old age (Tarnanas et al., 2014a,b), which has led to an increasing number of cognitive impairments in the elderly. Progressive decline in cognitive function leads to neuropsychological disorders such as Alzheimer’s disease (AD), including amnesic cognitive impairment in high-risk populations of AD (Tarnanas et al., 2015). Older people with cognitive impairments may not be able to take care of their own lives. They may lose their memory, the ability to navigate through spaces, and the ability to communicate with others. They may not be able to participate in daily activities such as boiling water, cooking, driving, and so on. Cognitive impairment in the family can be a major burden on the family. Children may not have the time and energy to take care of the sick elderly, and the elderly often need professional caregivers to look after them. For the elderly, damage to the brain’s nervous system, which causes cognitive impairment, may even be irreversible, meaning the disease is incurable. For a country, a growing number of elderly people’s cognitive aging will lead to more social problems.

However, if the disease can be diagnosed in the early stages of cognitive training and recovery, it can be gradually recovered. But it is difficult to use neuropsychological methods for diagnosis, and there is no effective method of cognitive training. In this context, the introduction to virtual reality (VR) technology provides a very viable way to explore more cognitive diagnosis and recovery programs to prevent and delay cognitive aging, to ensure the normal life of the elderly, and to maintain the enthusiasm and vitality of independent life for the elderly (Klimova, 2016).

#### Virtual Reality Technology

In recent years, VR technology has developed rapidly, and it is one of the most promising and challenging technologies in computer graphics. VR is a technology that uses computer technology and interactive devices (such as head mounted displays, handles, gloves, etc.) to artificially create an environment close to the real world, allowing one or more people to interact with the virtual environment and its 3D entities (Gatica-Rojas and Méndez-Rebolledo, 2014; Tan et al., 2019a,b).

At present, VR has been proposed to treat some neuropsychological diseases such as anxiety and fear. VR technology can intuitively provide patients with an almost real environment; patients can be placed at the center of things and get better treatment results. At the same time, VR can also be used for surgical training to provide doctors with a recyclable and ecologically efficient way to gain more training experience. VR can also be used for other medical purposes such as post-stroke intervention and treatment.

In VR, several sensory perceptions are mainly used, including vision, touch, and kinematics. In the process of interaction between human and virtual environment, the transmission of information must be real-time, and it can make corresponding responses to human actions in time so that people can operate it fluidly. The use of VR in the diagnosis and treatment of cognitive impairment can provide a fully controlled experimental environment for timely observation of memory, emotion, perception, motion control, and other aspects of the user (Tarnanas et al., 2014a,b).

The environment provided by VR for the diagnosis or treatment of cognitive impairment can be divided into three levels of systemic immersion: non-immersion, semi-immersion, and full immersion. The difference between these three levels is the reality of the reality simulation. The better the immersion, the more realistic the virtual environment is, the more realistic the participants will be (García-Betances et al., 2015). Different experimental tasks may require varying degrees of immersion, and non-immersion limits the risks of simulation disease and is more portable than a 3D environment. But a higher level of immersion would provide a more realistic environmental effect. VR technology has obvious advantages in the diagnosis and treatment of cognitive impairment. First, it can provide an ecologically efficient way to diagnose and treat patients (Allain et al., 2014). VR can measure a subject’s behavior in an ecologically efficient environment and place the subject in a virtual environment to provide the physician with a judgment of the condition (Rizzo et al., 2004). Second, the combination of VR and neuropsychology as a non-pharmacological approach can be performed before symptoms occur (where symptoms mean the conditions that occur in patients with cognitive impairment, such as forgetting, being unable to move at will, etc.), and this approach is non-invasive. Third, experimenting in a virtual environment can avoid the dangers caused by improper patient operation and provide a safer environment. When participants enter a virtual environment, however, it can cause discomfort, such as dizziness. But it is undeniable that it is a very effective method for cognitive diagnosis and treatment.

## Cognitive Diagnosis and Treatment With VR

In this section, some recent studies on cognitive diagnosis and treatment using VR technology are briefly classified according to their research objectives, and the main research methods and expected results are summarized. Table 1 summarizes the main features of these articles. The research objectives of these articles are mainly divided into two aspects: the first is to evaluate and diagnose cognition, and the second is to train and treat cognition.

**Table 1:** Recent virtual reality (VR)-based studies on cognitive diagnosis and treatment.

First author	Participants	Virtual environment	Target	Results
Godehard Weniger	29 aMCI, 29 healthy controls	VR-Maze, VR-Park	Patients with aMCI were evaluated for self-centered and hetero-ego-centered memory	VR technique can indicate spatial memory impairment in aMCI patients
G.Plancher	29 aMCI, 29 healthy controls	Virtual automobile environment	Use VR to describe the characteristics of episodic memory	Virtual environment can be used as a tool to characterize episodic memory
Francesca Morganti	26 AD, 26 healthy controls	VR-Maze, VR-Road Map	Using VR technology, we study the transition from heterosexual space to self-space in early AD	In the early stages of AD, there were shortcomings in the planning of space missions
Silvia Serino	15 healthy controls, 15 aMCI, 15 early-AD	Virtual room	VR was used to detect the defects of egocentric and hetero-centric in early AD	AMCI patients and AD patients have defects in different aspects
Glen M. Doniger	125 adults with a family history of AD	Virtual supermarket	Cognitive motor training based on VR was given to middle-aged people with high risk of Alzheimer’s disease	Effectively improve cognitive ability
Déborah A. Foloppe	1 AD	Virtual kitchen	Enhance the autonomy of AD patients in cooking activities	Patients regain the ability to cook
Moffat Mathews	15 stroke patients	-	Recovery of prospective memory in stroke patients	Participants’ prospective memory improved well
Paul J.f. White	1 early-AD	Virtual buildings without landmarks	Neurocognitive therapy was performed in AD patients using VR navigation environment	Enhanced spatial cognition
Silvia Serino	20 AD, 8 healthy controls	Virtual city	Enhancing mental frame synchronization in AD patients	Effective improvement

### Research on Cognitive Diagnosis

Godehard Weniger’s studies assessed self-centered and hetero-ego-centered memory in individuals with amnesic mild cognitive impairment (aMCI) through VR. A total of 29 patients with aMCI and 29 healthy controls were enrolled in the study. In this experiment, a virtual maze of self-centered memories was set up, and a virtual park of hetero-ego-centered memories was set up. This study demonstrates the feasibility of VR techniques to study spatial memory deficits in aMCI patients, and the future direction of its use is to be able to predict the transition from MCI to AD and from normal to MCI (Weniger et al., 2011).

Plancher et al. (2012) used VR in aMCI and AD to study the characteristics of episodic memory. A total of 21 healthy elderly people, 15 aMCI patients, and 15 AD patients were selected to complete the task. Two virtual environments were designed: one for participants to act as drivers of virtual vehicles and the other for participants to act as passengers of virtual vehicles. The patient’s recall and identification of central information (i.e., environmental elements), contextual information (i.e., time, egocentric and distribution center spatial information), and the overall combined qualities were assessed. In this study, the performance of these participants was found to be consistent with atrophy of the hippocampus. The performance of AD patients was the worst. Spatial distribution memory assessment was particularly useful for distinguishing between aMCI patients and healthy older adults. Cognitive differences between the three groups can provide effective recommendations for the early diagnosis and rehabilitation of pathological aging, and they prove that the virtual environment can be used as a tool to characterize episodic memory.

Morganti et al. (2013) published a article in 2013 that used VR technology to study the early transition of AD from a heterosexual space to its own spatial capabilities. In the study, 26 early AD patients and 26 healthy seniors were assigned to two virtual environment tasks: a VR-Maze and a VR-Road Map, both of which provide different path finding processes. This study shows that in the early stage of AD, the planning of spatial tasks is insufficient, and, in the task of transforming heterosexual spatial memory into an effective path of path finding, the ability of spatial memory is insufficient.

In Serino et al.’s (2015) study, VR was used to detect the egocentric and hetero-centric defects of early AD. The study involved 15 patients with early AD, 15 aMCI patients, and 15 cognitively healthy subjects completing two tasks. In the first task, participants needed to indicate the location of the objects they remember on the real map, while in the second task they were invited to retrieve their locations from different starting points in the empty version of the same virtual room. Comparing the performances of the three groups of participants showed that aMCI patients had deficiencies in storing hetero-centric independent representational memories and AD patients had deficiencies in storing hetero-centric independent representational memories and their synchrony. This may reflect the selective flaws in spatial organization, and these findings provide a preliminary understanding of the cognitive basis of amnesic damage.

### The Goal Is to Study Cognitive and Training Therapy

Doniger et al.’s (2018) study of VR-based cognitive motor training for middle-aged people at high risk of AD used VR technology to intervene in middle-aged people with high risk of AD in a non-pharmaceutical manner, offering effective prevention possibilities for middle-aged asymptomatic but high-risk people. In this study, a total of 125 middle-aged people aged 40–65 years with a family history of AD participated in the study and were randomly divided into four groups. The first group performed cognitive stimulation on a treadmill, the second group performed the same task without a treadmill, the third group performed non-specific cognitive stimulation on a treadmill, and the fourth group did not receive training as a control group. After 3 months of training, they conducted a cognitive assessment and concluded that cognitive training can effectively improve cognitive ability.

Foloppe et al. (2018) used a case study to discuss how VR-based cognitive training can enhance the autonomy of AD patients in cooking activities. The study evaluated a 79-years-old AD patient who was trained in four cooking tasks in a non-immersive manner, and it showed that VR restored the patient’s ability to perform cooking activities.

Mathews et al. (2016) used a virtual environment to test prospective memory (PM) recovery in stroke patients. Using non-immersive VR techniques, the study of 15 stroke patients showed that participants’ PM was significantly improved by VR-based cognitive training.

White and Moussavi (2016) used a VR navigation (VRN) environment for neurocognitive therapy in AD patients. The study involved a 74-year-old man who was in the early stages of AD and was asked to perform navigation tasks in a virtual environment. The results showed that the cognitive ability of the participants was enhanced by the cognitive rehabilitation task.

Serino et al. (2017) conducted a new VR-based training approach to enhance psycho-frame synchronization of AD patients. “Psychological frame synchronization” refers to the specific cognitive process of transition between egocentric and hetero-egocentric. The project recruited eight healthy elderly people and 20 AD patients, of whom 10 were healthy. Ten AD patients performed navigation tasks in a virtual environment, and the other 10 AD patients served as controls. The results showed that the spatial memory of AD patients and healthy people were effectively improved.

## Conclusion

VR technology is a very effective tool for cognitive assessment and recovery. It enables researchers and doctors to monitor patients’ responses in real time, which traditional methods may not have permitted them to do. The interactivity of VR allows participants to use multiple senses for feedback to get more details. VR works in an ecologically efficient way and pages are safer. There is still room for improvement in the study of VR for cognitive diagnosis and recovery. In most studies, the number of participants was insufficient, which led to more uncontrollable factors in the experiment. Therefore, in an experiment, we need to recruit as many participants as possible to improve the participation of patients, better understand their needs, coordinate different services, and access participants in a long-term, timely manner to obtain experiments. Although much research has been done, more research is needed on the scale of its use in patients.

## Author Contributions

YL wrote this article. WT presented the major idea of this article. CC collected the research articles. CL presented the method of diagnosis and treatment of cognitive impairment in hospital. JY corrected the article. YZ presented the VR method in the diagnosis and treatment of cognitive impairment.

## Conflict of Interest

The authors declare that the research was conducted in the absence of any commercial or financial relationships that could be construed as a potential conflict of interest.

## References

[B1] Allain P. Foloppe D. A. Besnard J. Yamaguchi T. Etcharry-Bouyx F. Le Gall D.. (2014). Detecting everyday action deficits in Alzheimer’s disease using a nonimmersive virtual reality kitchen. J. Int. Neuropsychol. Soc. 20, 468–477. 10.1017/S1355617714000344 24785240

[B2] Anguera J. A. Boccanfuso J. Rintoul J. L. Al-Hashimi O. Gazzaley A. (2013). Video game training enhances cognitive control in older adults. Nature 501, 97–101. 10.1038/nature12486 24005416 PMC3983066

[B5] Doniger G. M. Beeri M. S. Bahar-Fuchs A. Gottlieb A. Tkachov A. Kenan H.. (2018). Virtual reality-based cognitive-motor training for middle-aged adults at high Alzheimer disease risk: a randomized controlled trial. Alzheimers. Dement. 4, 118–129. 10.1016/j.trci.2018.02.005 29955655 PMC6021455

[B6] Foloppe D. A. Richard P. Yamaguchi T. Etcharry-Bouyx F. Allain P. (2018). The potential of virtual reality-based training to enhance the functional autonomy of Alzheimer’s disease patients in cooking activities: a single case study. Neuropsychol. Rehabil. 28, 709–733. 10.1080/09602011.2015.1094394 26480838

[B7] García-Betances R. I. Arredondo Waldmeyer M. T. Fico G. Cabrera-Umpiérrez M. F. (2015). Corrigendum: a succinct overview of virtual reality technology use in Alzheimer’s disease. Front. Aging Neurosci. 7:235. 10.3389/fnagi.2015.00235 26696887 PMC4677601

[B8] Gatica-Rojas V. Méndez-Rebolledo G. (2014). Virtual reality interface devices in the reorganization of neural networks in the brain of patients with neurological diseases. Neural Regen. Res. 9, 888–896. 10.4103/1673-5374.131612 25206907 PMC4146258

[B3] Klimova B. (2016). Computer-based cognitive training in aging. Front. Aging Neurosci. 8:313. 10.3389/fnagi.2016.00313 28066236 PMC5168996

[B9] Mathews M. Mitrovic A. Ohlsson S. Holland J. McKinley A. (2016). A virtual reality environment for rehabilitation of prospective memory in stroke patients. Procedia Comput. Sci. 96, 7–15. 10.1016/j.procs.2016.08.081

[B10] Morganti F. Stefanini S. Riva G. (2013). From allo- to egocentric spatial ability in early Alzheimer’s disease: a study with virtual reality spatial tasks. Cogn. Neurosci. 4, 171–180. 10.1080/17588928.2013.854762 24251605

[B11] Plancher G. Tirard A. Gyselinck V. Nicolas S. Piolino P. (2012). Using virtual reality to characterize episodic memory profiles in amnesic mild cognitive impairment and Alzheimer disease: influence of active and passive encoding. Neuropsychologia 50, 592–602. 10.1016/j.neuropsychologia.2011.12.013 22261400

[B12] Rizzo A. A. Schultheis M. T. Kerns K. Mateer C. (2004). Analysis of assets for virtual reality applications in neuropsychology. Neuropsychol. Rehabil. 14, 207–239. 10.1080/09602010343000183

[B14] Serino S. Morganti F. Di Stefano F. Riva G. (2015). Detecting early egocentric and allocentric impairments deficits in Alzheimer’s disease: an experimental study with virtual reality. Front. Aging Neurosci. 7:88. 10.3389/fnagi.2015.00088 26042034 PMC4438252

[B15] Serino S. Pedroli E. Tuena C. De Leo G. Stramba-Badiale M. Goulene K.. (2017). A novel virtual reality-based training protocol for the enhancement of the “mental frame syncing” in individuals with Alzheimer’s disease: a development-of-concept trial. Front. Aging Neurosci. 9:240. 10.3389/fnagi.2017.00240 28798682 PMC5529401

[B16] Tan W. J. Kang Y. A. Dong Z. W. Chen C. Yin X. Su Y. (2019a). An approach to extraction midsagittal plane of skull from brain CT images for oral and maxillofacial surgery. IEEE Access 7, 118203–118217. 10.1109/access.2019.2920862

[B17] Tan W. J. Yuan Y. Chen A. Mao L. Ke Y. Lv X. (2019b). An approach for pulmonary vascular extraction from chest CT images. J. Healthc. Eng. 2019:9712970. 10.1155/2019/9712970 30800258 PMC6360062

[B18] Tarnanas I. Laskaris N. Tsolaki M. Muri R. Nef T. Mosimann U. P. (2015). On the comparison of a novel serious game and electroencephalography biomarkers for early dementia screening. Adv. Exp. Med. Biol. 821, 63–77. 10.1007/978-3-319-08939-3_11 25416111

[B19] Tarnanas I. Tsolaki M. Nef T. Müri R. M. Mosimann U. P. (2014a). Can a novel computerized cognitive screening test provide additional information for early detection of Alzheimer disease? Alzheimers Dement. 10, 790–798. 10.1016/j.jalz.2014.01.002 24656838

[B20] Tarnanas I. Tsolakis A. Tsolaki M. (2014b). “Assessing virtual reality environments as cognitive stimulation method for patients with MCI,” in Technologies of Inclusive Well-Being. Studies in Computational Intelligence, eds Brooks A. Brahnam S. Jain L. (Berlin, Heidelberg: Springer), 39–74.

[B21] Weniger G. Ruhleder M. Lange C. Wolf S. Irle E. (2011). Egocentric and allocentric memory as assessed by virtual reality in individuals with amnesic mild cognitive impairment. Neuropsychologia 49, 518–527. 10.1016/j.neuropsychologia.2010.12.031 21185847

[B22] White P. J. Moussavi Z. (2016). Neurocognitive treatment for a patient with Alzheimer’s disease using a virtual reality navigational environment. J. Exp. Neurosci. 10, 129–135. 10.4137/JEN.S40827 27840579 PMC5102253

